# Diffusion tensor imaging of neurocognitive profiles in a community cohort living in marginal housing

**DOI:** 10.1002/brb3.1233

**Published:** 2019-02-06

**Authors:** Kristina M. Gicas, Alex Cheng, Iris Rawtaer, Taylor S. Willi, William J. Panenka, Donna J. Lang, Geoff N. Smith, Fidel Vila‐Rodriguez, Olga Leonova, Chantelle J. Giesbrecht, Andrea A. Jones, Alasdair M. Barr, Ric M. Procyshyn, Tari Buchanan, G. William MacEwan, Wayne Su, Alexandra T. Vertinsky, Alexander Rauscher, Norm O’Rourke, Wendy Loken Thornton, Allen E. Thornton, William G. Honer

**Affiliations:** ^1^ Department of Psychology Simon Fraser University Burnaby Canada; ^2^ Department of Psychiatry University of British Columbia Vancover Canada; ^3^ Department of Radiology University of British Columbia Vancover Canada; ^4^ Department of Anesthesiology, Pharmacology and Therapeutics University of British Columbia Vancover Canada; ^5^ Department of Paediatrics University of British Columbia Vancover Canada; ^6^ Department of Public Health and Centre for Multidisciplinary Research in Aging University of the Negev Be’er Sheva Israel

**Keywords:** diffusion tensor imaging, multimorbidity, neurocognition, structural brain imaging, white matter

## Abstract

**Objective:**

We investigated white matter differences associated with distinct neurocognitive profiles derived from a large cohort of marginally housed persons with comorbid physical and mental illnesses. Our prior work identified three profile cluster groups: a high functioning group (Cluster 1), a low functioning group with relative strength in decision‐making (Cluster 3), and an intermediary group with a relative decision‐making weakness (Cluster 2). This study extends previous findings of cortical gray matter differences between these groups with evidence for putative neurodevelopmental abnormalities in the low cognitive functioning group (i.e., Cluster 3). We hypothesized that altered white matter diffusion would be associated with the lowest functioning neurocognitive profile and would be associated with previously observed gray matter differences.

**Method:**

Participants from a socially impoverished neighborhood in Vancouver, Canada underwent neurocognitive evaluation and neuroimaging. We performed Tract‐Based Spatial Statistics using diffusion tensor imaging data from 184 participants to examine whole‐brain differences in white matter microstructure between cluster analytically derived neurocognitive profiles, as well as unitary neurocognitive measures. Correlations between frontal gray and white matter were also examined.

**Results:**

Cluster 3 showed increased diffusion in predominately bilateral frontal and interhemisphere tracts (vs. Clusters 1 and 2), with relatively greater diffusion in the left hemisphere (vs. Cluster 1). Differences in radial diffusivity were more prominent compared with axial diffusivity. A weak association between regional frontal fractional anisotropy and previously defined abnormalities in gyrification was observed.

**Conclusions:**

In a socially marginalized sample, we established several patterns in the covariation of white matter diffusion and neurocognitive functioning. These patterns elucidate the neurobiological substrates and vulnerabilities that are apt to underlie functional impairments inherent to this complex and heterogeneous population.

## 1 INTRODUCTION

Living in marginal housing, such as single‐room occupancy (SRO) hotels is associated with serious consequences for health, well‐being, and psychosocial functioning (Vila‐Rodriguez et al., 2013). Co‐occurring polysubstance use, infectious diseases, and severe psychiatric illnesses are commonplace (Krausz et al., 2013; Vila‐Rodriguez et al., 2013) and entail significant costs, such as neurocognitive impairments (Gicas et al., 2017, 2014). These impairments may be central to the psychosocial dysfunction of marginalized persons. The extent to which multifactorial degradation of brain integrity underlies the neurocognitive impairment observed in marginalized persons warrants examination.

Previously we characterized extensive neurocognitive impairment of marginalized persons living in SRO hotels by grouping participants across multiple neurocognitive domains (attention, memory, executive functioning). Three subgroups with unique profiles of neurocognitive impairments were differentiated by cortical gyrification and thickness (Gicas et al., 2017, 2014). Our prior findings suggested that the poorest performing subgroup showed evidence of greater neurodevelopmental brain aberrations, whereas greater environmental risk exposures characterized persons in the other two subgroups. Nonetheless, our previous investigation was limited chiefly by its exclusive focus on cortical morphology. An analysis of white matter structure may prove complementary and fruitful given its vulnerability in many physical and mental health conditions that are endemic to marginalized populations. Understanding the neurological substrates for cognitive dysfunction is an important step in defining viable targets for specific rehabilitative interventions.

Diffusion tensor imaging (DTI) provides a sensitive measure of white matter tissue properties (Alexander, Lee, Lazar, & Field, 2007; Assaf & Pasternak, 2008). Fractional anisotropy (FA) is the most commonly used DTI metric, which reflects diffusion of water molecules restricted to one direction by the presence of axonal membranes and myelin sheaths (Assaf & Pasternak, 2008). Degradation of these tissue components leads to a decrease in FA values. Although FA is a non‐specific index of white matter abnormality, complementary information can be provided by the constituent components of the diffusion tensors. As demonstrated in animal models, diffusion of water molecules parallel to the axon is referred to as axial diffusivity (AD) and is thought to reflect axonal integrity, whereas diffusion perpendicular to the axon is referred to as radial diffusivity (RD) and is thought to reflect myelin integrity (Song et al., 2003, 2005).

Compared to healthy controls, significant white matter alterations in major frontal and interhemispheric tracts are reliably observed in users of stimulants (London, Kohno, Morales, & Ballad, 2015; Romero, Asensio, Palau, Sanchez, & Romero, 2010), opioids (Wollman et al., 2015), alcohol (Fortier et al., 2014), and polysubstances (Unterrainer et al., 2016), with longer durations of substance use correlated with greater white matter deficits (Ersche et al., 2012; Fortier et al., 2014; Wollman et al., 2015). Extensive white matter abnormalities have also been reported in schizophrenia (Ellison‐Wright & Bullmore, 2009; Samartzis, Dima, Fusar‐Poli, & Kyriakopoulos, 2014) and HIV infection (Holt, Kraft‐Terry, & Chang, 2012; Leite et al., 2013). Recent work has demonstrated that persons with comorbidities, such as HIV+psychostimulant users, also show significantly lower FA and higher diffusivity in select frontal and interhemispheric tracts, which correlated with several aspects of neurocognition (Tang et al., 2015). However, few studies have comprehensively studied white matter diffusion within multimorbid samples.

The aim of this study is to ascertain the extent to which white matter DTI variations differentiate previously derived and well validated neurocognitive subgroups (Gicas et al., 2017, 2014) in a sample of vulnerable persons primarily dwelling in unstable housing, such as SRO hotels. We used cluster analysis to subgroup individuals on the basis of similar profiles of neurocognitive strengths and weaknesses (Allen & Goldstein, 2013). This approach is optimal in a heterogeneous population because it allows us to identify more homogenous subgroups of persons characterized by distinct cognitive patterns and unique sets of neural and clinical vulnerabilities. Investigating these multidimensional structure‐function relationships is especially important in a marginally housed population due to varying combinations of co‐occurring illnesses that are likely to impact brain health and neurocognition in selective ways. We adopted a voxelwise whole‐brain approach using Tract‐Based Spatial Statistics (TBSS) to examine potential neurocognitive subgroup differences between major white matter tracts. This approach is highly suitable for a clinically heterogeneous population in which diffuse white matter abnormalities are likely to be prevalent and has the additional advantage of deriving mean DTI metrics from the center of white matter tracts, overcoming limits of the standard atlas‐based regional approach (Smith et al., 2006). We hypothesized that patterns of lower FA, with corresponding decreased AD and increased RD would be associated with the neurocognitive subgroup that exhibits the lowest functioning and greatest burden of physical and psychiatric illness (Gicas et al., 2017, 2014).

To better understand the nature and putative etiologies of hypothesized white matter differences, we examined associations between select major white matter tracts and cortical gray matter regions of interest (ROIs) previously found to differentiate the neurocognitive clusters (Gicas et al., 2017). Correspondence between white matter and gray matter alterations have been previously identified in schizophrenia patients, suggesting a possible common underlying pathophysiological mechanism (Liu et al., 2014). Given our previous findings of decreased cortical thickness and increased gyrification in the lowest functioning subgroup, we hypothesized poorer white matter DTI values will be associated with reduced regional cortical thickness and increased regional gyrification indices.

## 2 METHODS

### 2.1 Participants

Between November 2008 and November 2014, 371 participants were recruited from SRO hotels (*n* = 306) and the community courthouse (*n* = 65) located in an impoverished neighborhood ––the Downtown Eastside of Vancouver, BC. Participants were recruited as part of The Hotel Study described in detail elsewhere (Honer et al., 2017; Jones et al., 2015; Vila‐Rodriguez et al., 2013). Those with missing or invalid neurocognitive and DTI data were excluded, leaving a total of 185 participants. The inclusion/exclusion criteria are outlined in Figure S1. Participants 18 years of age or older were eligible for study inclusion if they were fluent in English and able to provide written informed consent. Participants received small honoraria. Sample characteristics are reported in Table 1. This study received ethics approvals from the Clinical Research Ethics Board of the University of British Columbia and the Simon Fraser University Office of Research Ethics.

**Table 1:** Sample characteristics by cluster

Independent Variable	Overall Sample	Cluster 1 (*n* = 55)	Cluster 2 (*n* = 72)	Cluster 3 (*n* = 58)	Cluster Comparisons
Age (years), *M *(*SD*)	43.1 (9.5)	44.0 (9.6)	42.8 (9.5)	42.5 (9.5)	ns
Education (years), *M *(*SD*)	10.4 (2.3)	11.1 (2.3)	10.2 (2.5)	9.9 (1.9)	C1>C3*
Monthly income (CAD), *M* (*SD*)	821 (362)	810 (452)	812 (32	844 (315)	ns
Duration living in DTES (years), *M (SD*)	7.9 (6.9)	6.9 (5.4)	8.2 (7.2)	8.6 (7.7)	ns
Charlson Comorbidity Index,* M* (*SD*)	3.4 (3.0)	3.2 (2.8)	3.3 (3.0)	3.7 (3.2)	ns
Sex (female), *n*(%)	39 (21.1)	5 (9.1)	24 (33.3)	10 (17.2)	C2>C1**, C3*
Ever homeless^a^, *n*(%)	133 (71.9)	42 (76.4)	49 (68.1)	43 (74.1)	ns
Ethnicity^b^, *n*(%)					
White	120 (64.9)	44 (80.0)	45 (62.5)	31 (53.4)	C1>C2*, C3**
First Nations	56 (30.3)	10 (18.1)	23 (32.0)	23 (39.7)	C1<C3*
Other	8 (4.3)	0 (0.0)	4 (5.6)	4 (6.9)	ns
Psychotic disorder, *n*(%)					
Schizophrenia spectrum	29 (15.7)	2 (3.6)	16 (22.2)	11 (19.0)	C1<C2**, C3*
Substance induced	32 (17.3)	6 (10.9)	10 (13.9)	12 (20.7)	ns
Other psychosis	23 (12.4)	8 (14.5)	6 (8.3)	9 (15.5)	ns
Substance dependence, *n*(%)					
Alcohol	32 (17.3)	27 (49.1)	33 (45.8)	32 (55.2)	ns
Cannabis	64 (34.6)	17 (30.9)	30 (41.7)	17 (29.3)	ns
Stimulant	156 (84.3)	46 (83.6)	65 (90.3)	45 (77.6)	C2>C3*
Opioid	76 (41.1)	24 (43.6)	33 (45.8)	19 (32.8)	ns

Note

*N = *185, unless otherwise specified. CAD: Canadian dollars; DTES: Downtown Eastside. Other psychosis includes: psychosis not otherwise specified, bipolar with psychosis, major depression with psychosis. Cluster comparisons were performed using Analysis of Variance with post‐hoc comparisons for continuous variables and chi‐square tests for categorical variables.

a *N* = 183.

b *N* = 184.

* *p* < 0.05,

** *p* < 0.005.

### 2.2 Neurocognitive and clinical assessments

Full details of the neurocognitive assessment are described elsewhere (Gicas et al., 2014). Trained research assistants administered a test battery that included the following measures: premorbid intellectual functioning (Wechsler Test of Adult Reading [WTAR FSIQ]; Wechsler, 2001), verbal learning and memory (total immediate recall score from Hopkins Verbal Learning Test Revised [HVLT‐R]; Brandt & Benedict, 2001), inhibition (Stroop color‐word subtest), sustained attention (signal detection [A'] from the Rapid Visual Information Processing [RVIP] subtest; Fray, Robbins, & Sahakian, 1996), mental flexibility (total adjusted errors score from the Intra‐Dimensional Extra‐Dimensional [IDED] subtest; Fray et al., 1996), and affective decision‐making (Iowa Gambling Task [IGT] net score; Bechara, Damasio, Damasio, & Anderson, 1994). An acculturation measure was administered to determine English language fluency.

To characterize the sample, details obtained from clinical assessments are included in Table 1. Clinical assessments were conducted by a neurologist, psychiatrist, and/or a research assistant separate from the neurocognitive testing. The Charlson Comorbidity Index was used to measure co‐occurring medical conditions according to the Charlson weighting scheme, with a point added for each decade of life over 40 years (Charlson, Pompei, Ales, & MacKenzie, 1987). Diagnoses of substance dependence and psychotic disorder were made by consensus using all available information including the Mini‐International Neuropsychiatric Interview (Sheehan et al., 1998), a mental status exam, and the Best Estimate Clinical Evaluation and Diagnosis (Endicott, 1988) adapted to the Diagnostic and Statistical Manual of Mental Disorders 4th edition criteria (American Psychiatric Association, 2000). A structured questionnaire was used to obtain sociodemographic information.

### 2.3 Neuroimaging acquisition and processing

Two identical DTI sequences per participant were acquired on a Philips Achieva 3.0 T scanner with an eight‐channel SENSE‐Head coil. Sequences were implemented at the start of the longitudinal study in 2008, and were maintained unchanged subsequently. The DTI scanning parameters were as follows: 32 gradient directions, acquisition matrix = 100 x 100 (reconstruction matrix = 112 x 112), field of view = 224 x 224 mm^3^, reconstructed voxel size = 2.0 x 2.0 X 2.2 mm, 70 slices with slice thickness = 2.2 mm (no gaps), TR/TE = 6,452/60 ms, flip angle = 90**°**, *b* factor = 700 s/mm^2^, total acquisition time = 3:45.8 min for each DTI sequence. Trained raters visually inspected all scans.

Exclusion parameters included DTI sequences containing greater than four gradient directions with artifacts, scans with motion artifact, or scans not completed proximal to neurocognitive testing (99% within 30 days, 1% within 1 year). Remaining volumes with artifacts were either removed or fixed with in‐house software. In participants with contraindications for scanning and in instances of equipment malfunction, DTI data were not available. Two DTI sequences were averaged after eddy current correction using the FMRIB's Diffusion Toolkit part of FMRIB's Software Library (FSL; Jenkinson, Beckmann, Behrens, Woolrich, & Smith, 2012). DTI fitting was run using a nonlinear least squares approach with shifted negative eigenvalues from 3D Slicer. Finally, a nonlinear registration method was used to co‐register DTI data with the JHU ICBM‐ DTI‐81 atlas (John Hopkins University International Consortium Brain Mapping; Mori et al., 2008).

Automatic cortical parcellation was conducted using FreeSurfer v5.1 software (https://www.nmr.mgh.harvard.edu/) to obtain regional measures of cortical thickness (CT) and local gyrification index (lGI), as described in our previous work (Gicas et al., 2017). Manual editing was performed on pial and white matter surfaces wherever segmentation errors occurred. Bilateral indices were created for the following CT and lGI ROIs as defined by the Desikan‐Killiany atlas (Desikan et al., 2006): medial orbitofrontal cortex (OFC), lateral OFC, anterior cingulate cortex, and entorhinal cortex.

### 2.4 Statistical analysis

#### 2.4.1 Neurocognitive clustering

In accordance with previous research (Gicas et al., 2017, 2014), we performed a k‐means cluster analysis with random seed points and specifying three groups using the Statistical Package for the Social Sciences 22.0. Neurocognitive variables used for clustering included scores from the WTAR, HVLT, Stroop, RVIP, IDED, and IGT. The IDED variable was log transformed due to significant skew and multiplied by −1 to maintain consistency of interpretation across scores. Participants were excluded if they had invalid or missing data on more than one neurocognitive measure, yielding a total of 299 participants retained for clustering. To control for the effects of age and education, all neurocognitive scores (except the WTAR FSIQ) were regressed on these demographic factors and the resultant standardized residuals (z‐scores) were entered as the dependent variables in the cluster analysis (Manly et al., 2011). We have previously applied this analytic approach to a subsample (*N* = 249) of these participants using a 2‐step cluster analysis, which was internally validated using a discriminant function analysis and a multiprofile multimethod correlation matrix (Gicas et al., 2014). A kappa coefficient was used to confirm that our current assignment of participants to clusters was consistent with our initial cluster analysis. Pearson correlation coefficients were computed to determine the degree of association between the various neurocognitive measures.

#### 2.4.2 Tract‐based spatial statistics

TBSS (Smith et al., 2006) from FSL and the randomize algorithm (Winkler, Ridgway, Webster, Smith, & Nichols, 2014) was used for comparisons of DTI metrics between the three clusters, with age and sex entered as covariates. Nonlinear coregistration of each FA image onto the JHU‐ICBM FA 1x1x1mm standard space was performed as a preliminary process for TBSS analysis. The average of all the FA images was built and skeletonized to form the mean FA skeleton, and thresholded at a standard FA value of 0.25. The average whole brain FA values were extracted and regressed on age and sex, and histograms were generated to examine the distribution of data within each group. Participant FA values were projected to the mean FA skeleton, along with AD and RD values using the same FA skeleton. Voxelwise statistics were then performed using the randomize command with the Threshold‐free Cluster Enhancement option applied. TBSS results were visualized using FSLview thresholded at *p* < 0.05, and overlaid with the JHU‐ICBM‐DTI‐81 white matter atlas to identify the regions that significantly differed between groups.

#### 2.4.3 Secondary TBSS analysis

In an exploratory follow‐up to the main TBSS group‐based analysis, we wished to examine whether unitary domains of neurocognitive functioning were uniquely associated with variation in whole brain white matter. To examine this, we conducted a series of six TBSS analyses using the same approach described above in section 2.4.2, except we entered a different continuous neurocognitive measure instead of the cluster grouping variable for each analysis. This enables us to examine correlations between each neurocognitive measure and white matter FA within each voxel across the entire brain. The neurocognitive variables used in this approach were identical to those submitted to the cluster analysis, and included the standardized residuals (z‐scores) for HVLT‐R, Stroop, RVIP A, IDED, and IGT. The WTAR FSIQ standard scores converted to z‐score units were also included. Age and sex were entered as covariates. Scatterplots of age‐ and sex‐adjusted mean whole brain FA values (derived from the TBSS skeleton) and each of the neurocognitive variables were visually inspected for outliers.

#### 2.4.4 Correlational analysis

Partial correlations, controlling for age and sex, were used to examine associations between select cortical gray matter and white matter tracts within the overall sample. We selected regions of cortical thickness and gyrification that were previously found to differentiate the clusters (Gicas et al., 2017), and paired these with the corresponding white matter tracts that are neuroanatomically linked to these cortical ROIs. Gray matter ROIs included the following: entorhinal cortex (lGI), lateral OFC (lGI), medial OFC (lGI, CT), and anterior cingulate cortex (CT). The corresponding white matter ROIs included FA and RD measurements for the anterior corona radiata, and superior longitudinal fasciculus. Eight partial correlations were conducted for each set of selected DTI metrics, and a Bonferroni correction was applied setting the critical alpha value to *p* < 0.006 (0.05/8). In follow‐up, significant correlations at the Bonferroni‐corrected level were analyzed within a moderation model using the SPSS PROCESS Macro v2.16.3 (Hayes, 2013) to determine whether the strength of the gray‐white matter correlations were significantly different across clusters (multicategorical moderator). Visual inspection of scatterplots and histograms of the residuals for the multiple regression model were conducted to identify possible outliers.

## 3 RESULTS

### 3.1 Cluster analysis

**Figure 1:**
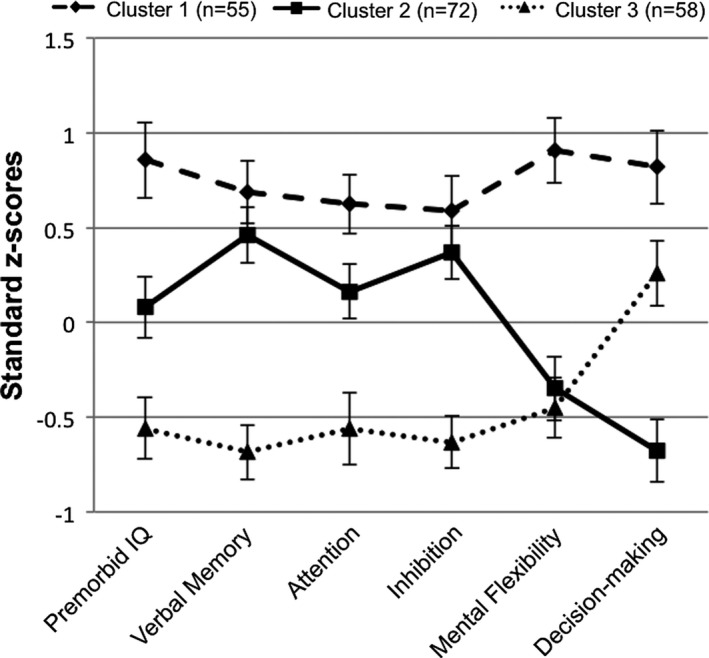
Neurocognitive profiles by cluster membership (*N* = 185). Error bars represent 95% confidence intervals

**Figure 2:**
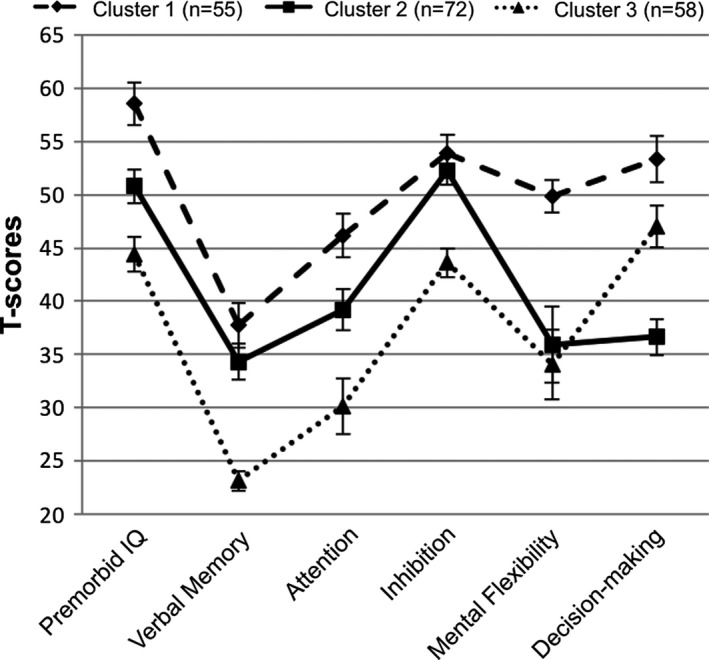
Demographically corrected neurocognitive profiles by cluster membership (*N* = 185). Errors bars represent 95% confidence intervals

As previously reported, there was excellent agreement between cluster assignment in our original analysis of 249 participants and in our current analysis (*κ* = 0.84; Gicas et al., 2017). Cluster 1 (*n* = 87; 29.1%) demonstrated the highest neurocognitive functioning across all domains, while Cluster 3 (*n* = 103; 34.4%) demonstrated the lowest functioning overall, with a relative strength in decision‐making. Cluster 2 (*n* = 109; 36.5%) exhibited neurocognitive abilities that were intermediary to Clusters 1 and 3, with a relative weakness in decision‐making. While a total of 299 participants had valid/complete neurocognitive data and were included in the cluster analysis, the neurocognitive profiles were constructed using the reduced sample with valid MRI data (*N* = 185) and are depicted in Figure 1. Profiles adjusted for age and education using normative test databases are displayed in Figure 2 for descriptive purposes. Small‐to‐moderate, positive correlations between all neurocognitive measures were observed (Table S1), suggesting that these are indexing relatively orthogonal domains of functioning.

### 3.2 Tract‐based spatial statistics

**Figure 3:**
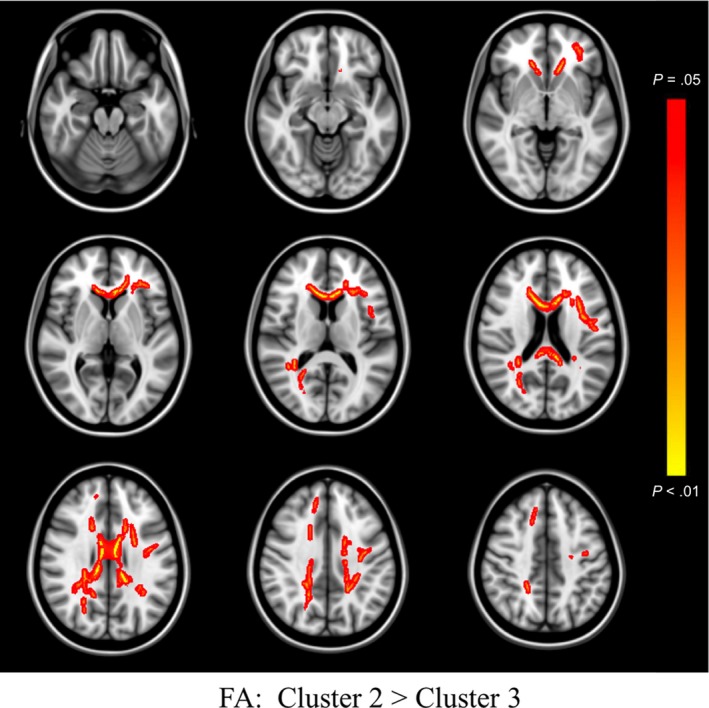
TBSS FA Differences Between Clusters. Colored regions (red to yellow) signify decreased fractional anisotropy (FA) in Cluster 3, relative to Cluster 2, at *p* < 0.05 (corrected for multiple comparisons). Images are presented in radiological space

**Figure 4:**
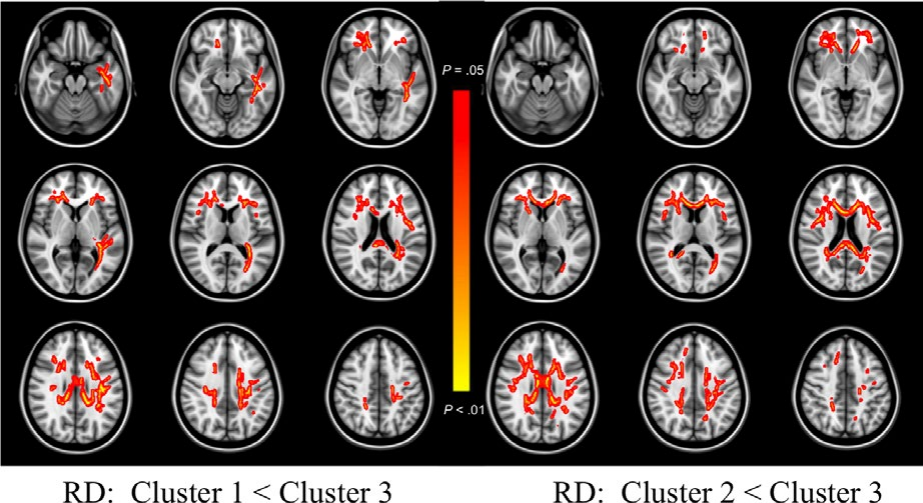
TBSS RD Differences Between Clusters. Colored regions (red to yellow) signify increased radial diffusivity (RD) in Cluster 3, relative to Cluster 1 (left‐sided panel) and Cluster 2 (right‐sided panel), at *p* < 0.05 (corrected for multiple comparisons). Images are presented in radiological space

**Figure 5:**
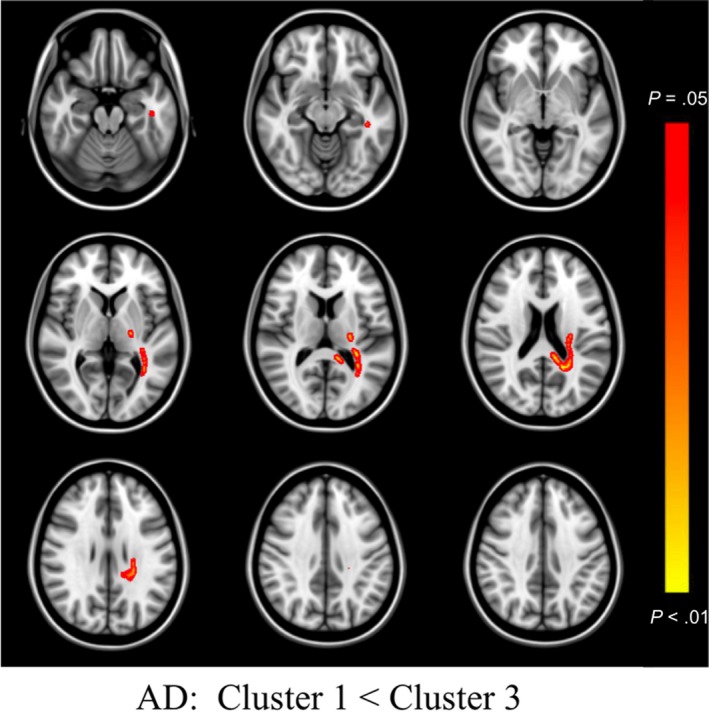
TBSS AD Differences Between Clusters. Colored regions (red to yellow) signify increased axial diffusivity (AD) in Cluster 3, relative to Cluster 1, at *p* < 0.05 (corrected for multiple comparisons). Images are presented in radiological space

Inspection of histograms of residualized FA values identified one case as an outlier and analyses were conducted with this case excluded (initially assigned to Cluster 3). When comparing Cluster 1 versus Cluster 3, differences in whole brain FA did not meet our threshold for statistical significance (*p* = 0.066). However, when the constituent components of the diffusion tensor were examined, greater RD (*p* = 0.015) was observed in frontal and interhemispheric regions bilaterally and was relatively greater within the left hemisphere, and greater AD (*p* = 0.030) was observed selectively in left posterior tracts and the splenium of the corpus callosum for Cluster 3. When compared to Cluster 2, Cluster 3 showed lower FA (*p* = 0.024), and greater RD (*p* = 0.016) largely in bilateral frontal and interhemispheric tracts, with no differences observed on AD (*p* = 0.111). All significant results are visualized in Figures 3, 4, 5. No areas of significantly different FA, RD, or AD were observed between Cluster 1 and Cluster 2. When analyses were re‐run with the outlier included, the pattern of findings largely remained the same, though effects became stronger, and a significant effect emerged for lower whole brain FA in Cluster 3 versus Cluster 1, and greater AD in Cluster 3 versus Cluster 2.

### 3.3 Secondary TBSS analysis

No significant correlations were observed between white matter FA and any of the neurocognitive measures at the adjusted *p* < 0.05 threshold.

### 3.4 Correlational analysis

No significant gray‐white matter associations were observed at the Bonferroni‐corrected alpha level (*p* < 0.006). At the conventional alpha level (*p *< 0.05), increased medial OFC gyrification was associated with decreased anterior corona radiata FA (*pr* = −0.18, *p* = 0.013), with increased anterior corona radiata RD (*pr* = 0.19, *p* = 0.011), and with decreased superior longitudinal fasciculus FA (*pr* = −0.16, *p* = 0.027). Results are summarized in Table 2. Given minimal cluster differences were identified for AD values, this metric was not further examined in our correlational analyses.

**Table 2:** Regional gray‐white matter partial correlations controlling for age and sex

Paired regions of interest	Partial correlation coefficient	*p*‐values
Entorhinal lGI – Cingulum		
FA	−0.098	0.190
RD	0.109	0.142
Medial orbitofrontal lGI – Anterior corona radiata		
FA	−0.184	**0.013**
RD	0.188	**0.011**
Medial orbitofrontal lGI – Superior longitudinal fasciculus		
FA	−0.164	**0.027**
RD	0.141	0.058
Lateral orbitofrontal lGI – Anterior corona radiata		
FA	−0.060	0.419
RD	0.105	0.157
Lateral orbitofrontal lGI – Superior longitudinal fasciculus		
FA	−0.030	0.687
RD	0.037	0.617
Medial orbitofrontal CT – Anterior corona radiata		
FA	0.067	0.371
RD	−0.084	0.258
Medial orbitofrontal CT – Superior longitudinal fasciculus		
FA	0.122	0.100
RD	−0.139	0.061
Anterior cingulate CT – Cingulum		
FA	−0.108	0.147
RD	0.077	0.301

Note

*N = *184. Bold text denotes significance at the uncorrected alpha level (*p* <0.05). lGI = local gyrification index; FA = fractional anisotropy; RD = radial diffusivity; CT = cortical thickness.

Three separate moderation analyses were conducted for each of the significant partial correlations to determine if the gray‐white matter associations differed by cluster membership. The interaction between neurocognitive clusters and medial OFC gyrification did not account for a significant amount of variability in anterior corona radiata FA (*F* = 0.43, *p* = 0.653), RD (*F* = 0.48, *p* = 0.619), or superior longitudinal fasciculus FA (*F* = 0.31, *p* = 0.732).

### 3.5 Summary

Given the complex nature of this sample, we have opted to summarize the neurocognitive cluster findings to date. In Table 3 we present the results from this study as well as findings from our most recent study (Gicas et al., 2017) to provide greater context for the ensuing discussion.

**Table 3:** Full summary descriptions of neurocognitive clusters

Descriptor	Cluster 1	Cluster 2	Cluster 3
Neurocognition	*Higher* functioning overall.	*Intermediate* functioning, relative weakness in decision‐making.	*Lower* functioning, relative strength in decision‐making.
White Matter	*Increased* white matter microstructure, particularly in the left hemisphere.	*Increased* white matter microstructure, particularly in frontal and interhemispheric tracts.	*Decreased *white matter microstructure in bilateral frontal and interhemispheric regions.
Cortical Gray Matter	*Increased* cortical thickness in anterior cingulate and medial orbitofrontal regions (only in persons 50+ years).	*Decreased* cortical thickness in anterior cingulate and medial orbitofrontal regions (only in persons 50+ years).	*Increased* gyrification in the entorhinal and orbitofrontal regions. *Decreased* cortical thickness in anterior cingulate and medial orbitofrontal regions (only in persons 50 + years).
Risk Factors for Impairment	*Higher* years of education. *Lower* rates of MRI pathology (stroke, aneurysm).	*Greater* proportion of females. *Higher *rate of stimulant dependence.	*Greater* number of negative symptoms and neurological soft signs. *Higher* rate of schizophrenia and history of special education. *Lower* rates of opioid dependence and childhood abuse.

## 4 DISCUSSION

We observed evidence for significant white matter variations within a large marginally housed sample of adults with physical and mental illness comorbidities. We compared subgroups that were each characterized by a unique neurocognitive profile and relatively different rates of substance use, viral infection, and psychiatric illness (Gicas et al., 2017, 2014). Although we do not present a neurocognitive control group, normative profiles suggest that Cluster 1 exhibits relatively intact neurocognitive functioning, with the exception of memory (see Figure 2). Therefore, Cluster 1 may be considered a reasonable benchmark for interpreting group differences on brain structure as they relate to complex attention and executive functioning processes (i.e., neurocognitive domains that were considered within normal limits for Cluster 1).

In this study, we found that greater white matter alterations were consistently associated with the lower neurocognitive functioning group (Cluster 3) as hypothesized. The whole brain voxelwise analysis (TBSS) revealed a pattern of bilateral and predominately frontal and interhemispheric reductions in FA and increases in RD for Cluster 3 (vs. Cluster 2), whereas a similar pattern with a stronger left hemisphere effect was observed for Cluster 3 compared to Cluster 1, but only for RD. Select AD increases in the left posterior regions was also observed in Cluster 3 (vs. Cluster 1). Secondary analyses of unitary neurocognitive measures did not show any significant associations with whole brain white matter variation. Within the overall sample, weak associations between the medial OFC gyrification and white matter diffusion were evident. This may point towards a common etiology of morphological abnormalities within this frontal subregion and warrants further investigation.

As hypothesized, diffuse bilateral white matter differences, particularly in frontal and interhemispheric tracts, were observed in relation to Cluster 3. This pattern fits well with Cluster 3’s overall profile of lower neurocognitive functioning across domains relative to other groups within the sample, and with the substantial normative impairments in attention and mental flexibility. Indeed, global white matter microstructural integrity is fundamental to general cognitive functioning, facilitating rapid transmission, and integration of information from distributed networks (Penke et al., 2012). Our current findings for Cluster 3 of significant impairment in attention, memory, and mental flexibility, in conjunction with altered frontal and interhemispheric white matter microstructure suggests a possible contributory role of fronto‐subcortical circuitry in disrupted cognition. In particular, our findings implicate the dorsolateral prefrontal loop, which begins near the lateral surface of the anterior portion of the frontal lobe and projects to the basal ganglia (caudate and globus pallidus), then to the thalamus before routing information back to the dorsolateral prefrontal cortex (Bonelli & Cummings, 2007). The dorsolateral prefrontal cortex is closely linked with attentional control processes that facilitate sustained attention, holding and manipulating information in mind, and being able to flexibly shift attention between tasks (Stuss & Levine, 2002). Additionally, connectivity between the medial OFC and anterior cingulate cortex have been shown to play an important role in prefrontal cortex networks that support complex attentional‐control processes required for higher order cognition (Ohtani et al., 2017). Prefrontal circuitry dysfunction has been linked with cognitive deficits in schizophrenia (Lewis & González‐Burgos, 2008) and drug addiction (Koob & Volkow, 2010), two prominent characteristics of the current sample.

In addition to a fronto‐subcortical pattern, we also observed a subtler pattern of relatively greater white matter differences in the left hemisphere of Cluster 3. This subgroup was previously defined as having a higher burden of psychiatric illness compared to the other groups, including a higher proportion of persons with a schizophrenia diagnosis, more negative symptoms, and greater neurological soft signs (Gicas et al., 2017), and collectively these markers may implicate risk for left hemisphere alterations in the current sample. Left lateralization of white matter abnormalities is commonly noted in schizophrenia patient populations (Ellison‐Wright & Bullmore, 2009), and greater negative symptom severity has been linked to reduced FA in the majority of left‐sided white matter tracts (Asami et al., 2014). Furthermore, in a subset of the Hotel Study participants, we have previously reported reduced FA in frontal and interhemispheric tracts in persons with comorbid cocaine dependence and substance induced psychosis compared to those with cocaine dependence alone, with relatively greater left‐sided FA reductions (Willi et al., 2017). In healthy developing persons, there is a pattern of higher FA in the right hemisphere compared to the left, particularly in the right superior and inferior longitudinal fasciculi (Uda et al., 2015), which may suggest a vulnerability to reductions in left hemisphere white matter microstructural integrity with deviations in development.

While differences in AD values between the neurocognitive clusters were minimal, the finding of selective left hemisphere increases in AD associated with the poorest functioning subgroup was somewhat surprising and in contrast to our initial expectation, given that *decreased* AD is typically thought to be reflective of axonal injury (Song et al., 2003). However, it has been suggested that variations in AD may reflect decrease in axonal membrane density or reduction in number of axons and/or axonal spacing (Sen & Basser, 2005). In animal models of ischemia, Song et al. (2003) observed an initial decrease in AD followed by a trend toward increasing values with concurrent normalization of mean diffusivity, and this was interpreted to be a function of tissue loss. Alternatively, AD and RD tend to decrease during early development (Kumar, Nguyen, Macey, Woo, & Harper, 2012) and the relatively higher values observed in Cluster 3 may, in part, reflect deviations or arrest of normative white matter developmental trajectories, in keeping with our neurodevelopmental conceptualization of Cluster 3 (Gicas et al., 2017). While demyelination may play a prominent role in degradation of white matter microstructure in the current sample, some degree of axonal alteration is also likely to be contributory. The ostensible dynamic nature of these DTI parameters necessitate longitudinal DTI studies to better understand the relationship between these measures and underlying tissue architecture.

Our secondary analyses did not reveal any association between unitary neurocognitive measures and whole brain white matter variation. The discrepancy between our group‐based findings and the secondary analyses might be explained by differences in what a single score versus multiple scores captures in this multimorbid sample. For example, a poor memory score may be related to frontal subcortical dysfunction that impacts learning new information or dysfunction of medial temporal lobe regions that are required for encoding, consolidation, and retrieval processes. Thus, variation in memory scores alone may be associated with multiple discrete neural substrates across participants that arise from a host of idiosyncratic disease and developmental processes. Consequently, when select domains are individually inspected, associations between a given neurocognitive domain and whole white matter variations are potentially attenuated in our heterogenous sample. On the other hand, considering memory scores in conjunction with co‐variation in other domain‐specific scores maximizes within‐cluster neurocognitive homogeneity as well as between‐cluster profile differences, providing a more refined lens for identifying the shared neural phenotypes of each cluster that are related to differences in each cluster's specific neurocognitive profile.

Evaluating gray and white matter associations can provide important information about anatomical connectivity and may highlight regions with a common etiology of structural alterations. We observed modest correlations between greater medial OFC gyrification and altered diffusion of the anterior corona radiata and superior longitudinal fasciculus ––tracts with varied projections to frontal regions, though this did not survive a correction for multiple comparisons. While we cannot directly address the causal mechanisms implicated in gray‐white matter associations, it is possible that focal alterations in gyrification may result in changes to proximal white matter tracts as a function of deviation in typically coordinated patterns of development. Associations between cortical gray matter and white matter diffusion have been observed in frontal regions in schizophrenia (Liu et al., 2014) and autism spectrum disorder (Ecker et al., 2016), suggestive of a role for aberrant neurodevelopmental trajectories. A plausible alternative is that gray‐white matter covariation could be driven by transneuronal degeneration, whereby altered white matter pathways result in disrupted input/output, which in turn impacts upon cortical morphology. While we controlled for the effects of age in our partial correlations, it should also be cautioned that FA is apt to be more sensitive to the effects of aging and various environmental risk exposures compared to gyrification and this may complicate our interpretation of white matter‐gyrification associations.

Limitations of this study must be considered. First, we observed differences in white matter between well‐defined neurocognitive groups, but we did not have a healthy comparison group to determine the extent to which the white matter signal is truly abnormal. However, the relatively intact attention and executive profile of Cluster 1, and generally lower burden of illness overall, suggest that it can serve as a useful comparison for understanding group differences related to frontal region structure and function. Second, given the complexity and heterogeneity of this population, we cannot determine the causal contributors to the patterns of altered diffusion observed in Cluster 3. The relatively higher rates of psychiatric illness and differences in gyrification within this subgroup point toward neurobiological vulnerabilities of possible neurodevelopmental origin (Gicas et al., 2017), and these in turn may interact with differential risk exposure (substance use, viral infection) to confer an increased risk of white matter degradation. For example, there is evidence that white matter abnormalities may both predate development of addiction and follow from stimulant exposure (Ersche et al., 2012). There is also evidence for cumulative white matter damage in alcohol users with HIV infection (Pfefferbaum, Rosenbloom, Adalsteinsson, & Sullivan, 2007). Interactions between developmental and environmental factors, and their impact on white matter would be best examined in follow‐up with longitudinal models to address temporal associations. Third, it is important to note that the extent to which these findings generalize to other marginalized populations is unclear.

There are also inherent technological limitations of DTI that should be acknowledged. One of the main drawbacks is the partial volume effect, which occurs when anisotropy is artificially lowered due to fibers crossing or when tissues are mixed at the white matter/gray matter boundary (Assaf & Pasternak, 2008). Attempts to mitigate this problem include thresholding the FA values between 0.2 and 0.3 (0.25 in the current study) and using TBSS to create a mean skeleton that generates FA values from tract centers, thus avoiding standard smoothing and alignment procedures that increase partial volume (Smith et al., 2006). The other main drawback of DTI is the assumption that diffusion of water molecules in white matter follows a normal Gaussian distribution, which is apt to be violated under conditions of abnormal white matter (Assaf & Pasternak, 2008). Again, TBSS addresses this issue by demonstrating that normality is improved when FA values are taken from tract centers (Smith et al., 2006). Last, interpretations regarding the underlying tissue pathology should be taken with caution. It has been demonstrated that the eigenvalues of the diffusion tensor (in other words “axial” and “radial” diffusivities) can be influenced by eigenvector rotation, which varies across conditions, such as in regions of partial volume for example (Wheeler‐Kingshott & Cercignani, 2009). Therefore, interpretations regarding DTI parameters as measures of white matter “integrity” may be misleading, and use of such terminology should be avoided (Jones, Knosche, & Turner, 2013). Despite these technological limitations, DTI measures are highly robust in identifying white matter abnormalities that are associated with significant neurocognitive consequences (Marquez de la Plata et al., 2011).

This study provides an important characterization of white matter DTI abnormalities in a marginalized sample with physical and mental illness comorbidities. The differential patterns of white matter diffusion across the three subgroups illuminate anatomical substrates of profiles of neurocognitive dysfunction and provide clues as to the neurobiological vulnerabilities that may modify structure‐function relationships. Understanding white matter variations can have useful clinical applications. For example, better white matter DTI measures at the start of treatment for cocaine dependence (Xu et al., 2010) and alcohol dependence (Sorg et al., 2012) has been linked with better treatment outcomes. Overall, such findings highlight the potential role of white matter to inform the degree and type of intervention required to maximize functional recovery. Ideally, future work will also focus on developmental and environmental risk factors that contribute to white matter alterations to inform preventative interventions.

## CONFLICT OF INTEREST

Dr. Honer has received consulting fees or sat on paid advisory boards for In Silico, Otsuka/Lundbeck, Newron, AlphaSights, the Centre for Drug Research and Development, and the Canadian Agency for Drugs and Technology in Health. He was additionally supported by the Jack Bell Chair in Schizophrenia. Dr. Vila‐Rodriguez received research support from Canadian Institutes of Health Research (CIHR), Brain Canada, Michael Smith Foundation for Health Research, and Vancouver Coastal Health Research Institute; received in‐kind equipment support for this investigator‐initiated trial from MagVenture; and has been on an advisory board for Janssen. Dr. Procyshyn has been a member of the following advisory boards in the past 3 years: Janssen, Lundbeck, and Otsuka; a member of the following speaker's bureaus in the past 3 years: Janssen, Lundbeck, and Otsuka; and received grants from the Canadian Institutes of Health Research. Dr. MacEwan has received consulting fees or sat on paid advisory boards for: Apotex, AstraZeneca, BMS, Janssen, Lundbeck, Otsuka, Pfizer, and Sunovion. He also received fees for lectures sponsored by AstraZeneca, BMS, Janssen, Otsuka, and Eli Lilly, and has received grants from Janssen Pharmaceuticals. Dr. Thornton has received grants from the Canadian Institutes of Health Research (CIHR) and the William and Ada Isabelle Steel Fund. Drs. Gicas, Rawtaer, Panenka, Lang, Smith, Leonova, Giesbrecht, Barr, Vertinsky, Rauscher, O'Rourke, and WL Thornton; and Mr(s). Cheng, Willi, Jones, Buchanan, and Su have no conflicts of interest to declare.

## AUTHORS CONTRIBUTION

KMG, NO, WJLT, AET, and WGH contributed to study design, data analysis and interpretation, and drafting of the manuscript. All other co‐authors contributed to data acquisition and/or data analysis and interpretation, and critically revised manuscript for intellectual content.
