# Supplementary figures and images for: Diffusion tensor imaging of neurocognitive profiles in a community cohort living in marginal housing
Source: Brain Behav. 2019 Feb 6;9(3):e01233. doi: 10.1002/brb3.1233 (PMC6422717; doi:10.1002/brb3.1233)

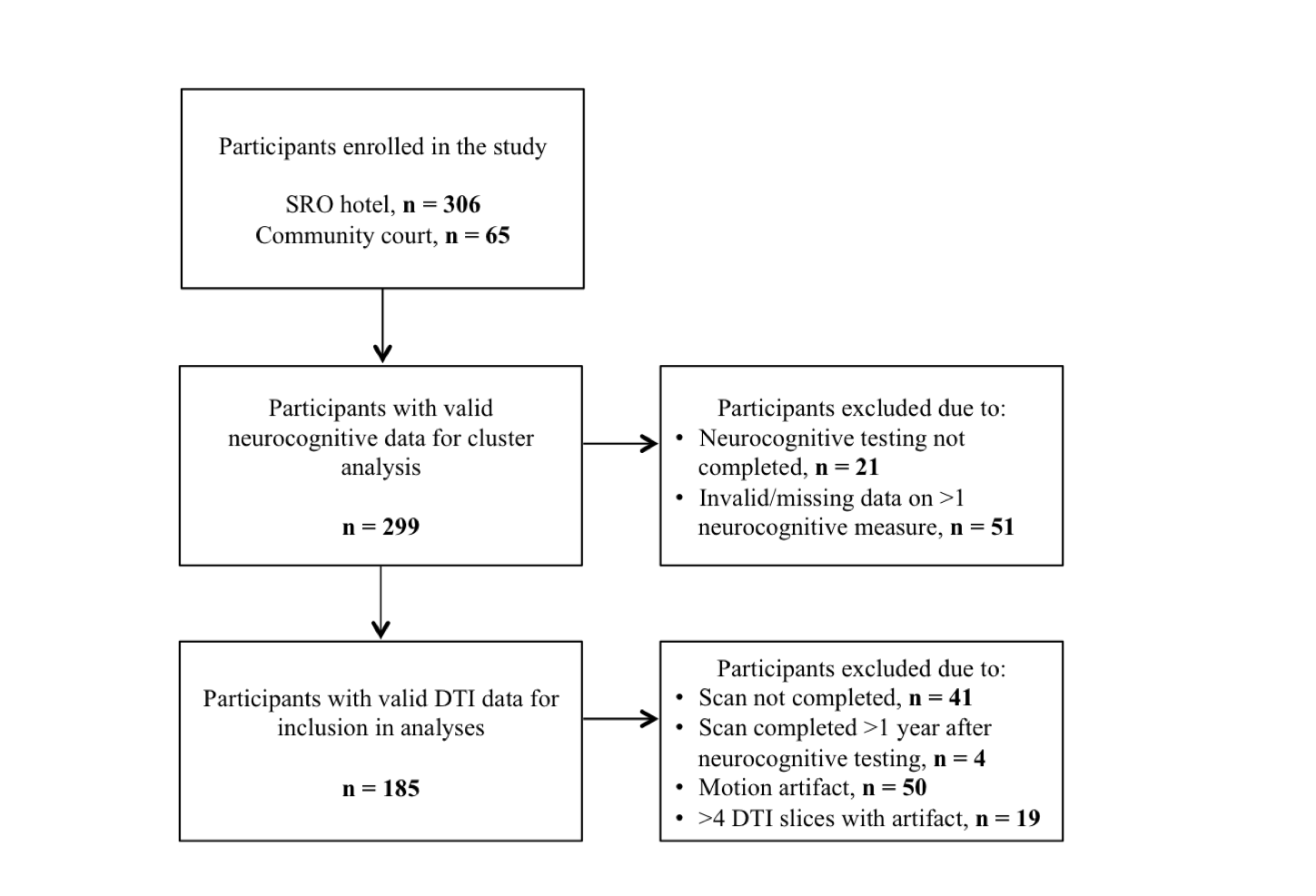

Supplement: Supplementary file 1 [file BRB3-9-e01233-s001.png]
