# Supplementary material for: Diffusion tensor imaging of neurocognitive profiles in a community cohort living in marginal housing
Source: Brain Behav. 2019 Feb 6;9(3):e01233. doi: 10.1002/brb3.1233 (PMC6422717; doi:10.1002/brb3.1233)
Supplement: Supplementary file 2 [file BRB3-9-e01233-s002.docx]

Table S1. Correlations Between Neurocognitive Variables

|  | Premorbid FSIQ | Verbal Memory | Attention | Inhibition | Mental Flexibility | Decision-Making |
| --- | --- | --- | --- | --- | --- | --- |
| Premorbid FSIQ |  | .301** | .264** | .244** | .233** | .076 |
| Verbal Memory |  |  | .361** | .283** | .170* | .047 |
| Attention |  |  |  | .389** | .310** | .093 |
| Inhibition |  |  |  |  | .174* | -.002 |
| Mental Flexibility |  |  |  |  |  | .235** |
| Decision-Making |  |  |  |  |  |  |

*Note.* Neurocognitive scores adjusted for age and education.

**p* < .05; ***p* < .01
